# The Ebola virus soluble glycoprotein contributes to viral pathogenesis by activating the MAP kinase signaling pathway

**DOI:** 10.1371/journal.ppat.1009937

**Published:** 2021-09-16

**Authors:** Wakako Furuyama, Kyle Shifflett, Heinz Feldmann, Andrea Marzi

**Affiliations:** Laboratory of Virology, Division of Intramural Research, National Institute of Allergy and Infectious Diseases, National Institutes of Health, Rocky Mountain Laboratories, Hamilton, Montana, United States of America; University of Texas Medical Branch / Galveston National Laboratory, UNITED STATES

## Abstract

Ebola virus (EBOV) expresses three different glycoproteins (GPs) from its GP gene. The primary product, soluble GP (sGP), is secreted in abundance during infection. EBOV sGP has been discussed as a potential pathogenicity factor, however, little is known regarding its functional role. Here, we analyzed the role of sGP *in vitro* and *in vivo*. We show that EBOV sGP has two different functions that contribute to infectivity in tissue culture. EBOV sGP increases the uptake of virus particles into late endosomes in HEK293 cells, and it activates the mitogen-activated protein kinase (MAPK) signaling pathway leading to increased viral replication in Huh7 cells. Furthermore, we analyzed the role of EBOV sGP on pathogenicity using a well-established mouse model. We found an sGP-dependent significant titer increase of EBOV in the liver of infected animals. These results provide new mechanistic insights into EBOV pathogenicity and highlight EBOV sGP as a possible therapeutic target.

## Author summary

Since its discovery in 1976, Ebola virus (EBOV) has caused infrequent outbreaks of hemorrhagic disease in Africa. The virus’ replication cycle has been well-characterized in cell culture using natural isolates and reverse genetics systems. For many EBOV proteins the key functions have been defined, however, the role the primary products of the glycoprotein (GP) gene, soluble GP (sGP), is not well understood. Our studies focused on sGP’s impact on different stages in the viral life cycle. While sGP increased the uptake of EBOV particles into late endosomes in a human kidney-derived cell line, it activated the mitogen-activated protein kinase signaling pathway leading to increased viral replication in a human liver-derived cell line. Analysis of sGP treatment in the well-established mouse model for EBOV infection demonstrated that sGP treatment indeed increases virus replication in key target tissues like liver and spleen. This data suggests a contribution of sGP to EBOV pathogenicity and identifies it as a new target for therapeutic approaches.

## Introduction

Ebolaviruses are highly pathogenic viruses with human case fatality rates of up to 90% [1]. As best exemplified by the Ebola virus (EBOV) epidemic in West Africa, ebolaviruses pose a significant public health concern. Six distinct species are known in the genus *Ebolavirus*; *Zaire ebolavirus*, *Sudan ebolavirus*, *Taï Forest ebolavirus*, *Bundibugyo ebolavirus*, *Bombali ebolavirus*, and *Reston ebolavirus*, represented by Ebola virus (EBOV), Sudan virus, Taï Forest virus, Bundibugyo virus, Bombali virus, and Reston virus (RESTV), respectively [2]. Among those EBOV is the most pathogenic one, whereas RESTV has not been associated with human disease [1].

Ebolaviruses express three different proteins from their glycoprotein (GP) gene, expression of which is partially controlled by transcriptional editing at a site of 7 uridine residues (7U)[3–5]. When the viral polymerase encounters this editing site, in 70–80% no transcriptional editing occurs resulting in a primary 7U transcript (wildtype; wt), which leads to the production of the soluble GP (sGP). In the remaining 20–30% editing occurs resulting mainly in 8U transcripts encoding the full-length transmembrane GP, which is expressed on the virion surface, or to a low frequency in 9U transcripts encoding the small sGP (ssGP). Full-length GP is essential for virus replication as it mediates attachment and entry into host cells [6,7]; in contrast, no definite function has been described yet for sGP and ssGP. EBOV entry is initiated by GP interacting with attachment factors, followed by internalization of the virus particle into cells largely via macropinocytosis [8–12]. In the late endosome, EBOV GP is cleaved by host proteases and binds to its receptor, Niemann-Pick C1 (NPC1), leading to membrane fusion [13,14]. The main product transcribed from the GP gene, sGP, is found in large quantities in the serum of patients [5]. Since sGP is the primary product of the GP gene and shares part of its primary protein sequence with GP, it has been discussed as a potential antibody decoy [15]. Despite the abundance of sGP during infection, little is known regarding its actual functional role.

In this study, we investigated the effect of sGP on EBOV infectivity and pathogenicity. We show that EBOV sGP but not RESTV sGP increases the infectivity of EBOV in two cell lines and in the mouse model. This is the first report demonstrating that EBOV sGP activates the mitogen-activated protein kinase (MAPK) signaling pathway resulting in increased EBOV replication.

## Results

### EBOV sGP contributes to virus uptake in HEK293 cells

**Fig 1:**
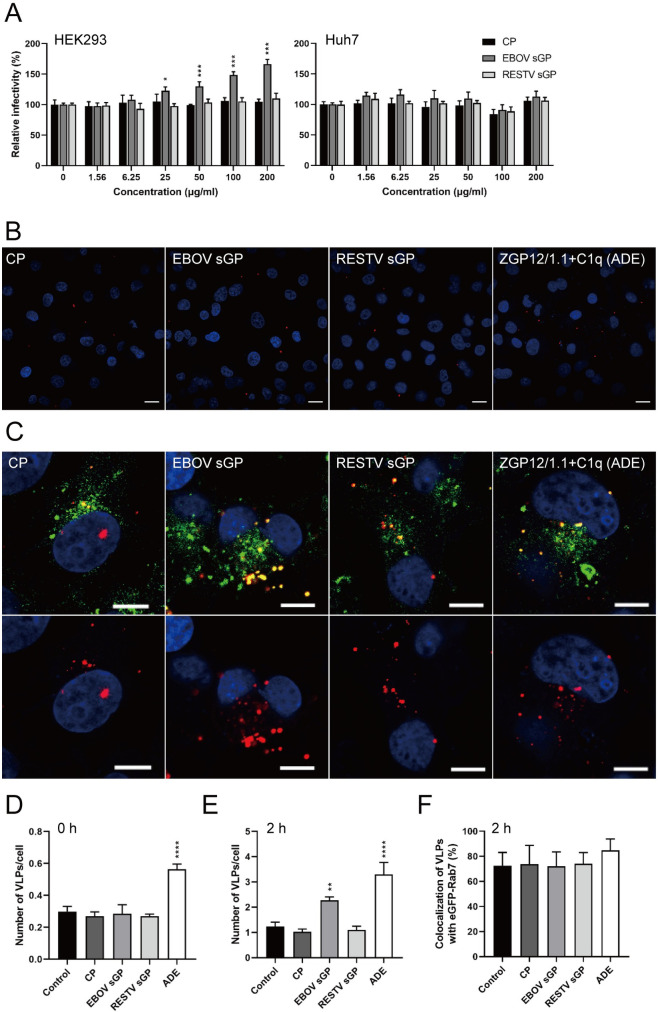
EBOV sGP enhanced the VLP uptake into endosomes in HEK293 cells. (**A**) VSV-EBOV GP pseudoparticles (100 IU/well) were inoculated in HEK293 or Huh7 cells in the presence of the indicated concentrations of EBOV sGP, RESTV sGP, or the equivalent amount of CP. After 24 h, the relative infectivity was calculated by setting the value of each infected cell line without protein to 100%. (**B, C**) DiI-labeled VLPs were inoculated with medium alone (control), 100 μg/ml EBOV sGP, RESTV sGP, or equivalent amount of CP into HEK293-Rab7 GFP cells. EBOV GP-specific antibody ZGP12/1.1 (10 μg/ml) with C1q (20 μg/ml) (ADE) was used as a positive control. VLPs (red) on the cell surface at 0 h (**B**), VLPs (red) and eGFP-Rab7 (green) in the cytoplasm at 2 h (**C**) after adsorption were monitored by confocal laser scanning microscopy. (**C**) Top panels show the merged image; bottom panels highlight VLPs. (**B, C**) Scale bars represent 10 μm. Cell nuclei are visualized with DAPI (blue). (**D**) Number of VLPs on the cell surface, (**E**) VLPs taken up into the cells, and (**F**) the colocalization of VLPs (DiI) with the eGFP-Rab7 signal. (**A, D-F**) The mean and standard deviation of three independent experiments are shown. Statistically significant differences as determined by one-way ANOVA are indicated as ****p < 0.0001, ***p < 0.001, **p < 0.01, and *p < 0.05.

Analysis of the effect of sGP on viral entry was performed using replication-incompetent vesicular stomatitis virus (VSV) pseudotyped with EBOV GP (VSV-EBOV GP)[7]. The system is well-established and has been used in previous studies investigating EBOV GP-mediated entry [7]. In addition, we produced recombinant EBOV and RESTV sGP in mammalian cells and concentrated it as described previously [16]. We infected HEK293 and Huh7 cells with VSV-EBOV GP in the presence of EBOV sGP, RESTV sGP, and a control protein (CP). Only EBOV sGP increased the infectivity of the pseudotyped virus in a dose dependent manner in HEK293 cells (Fig 1A left panel). However, there was no observed difference in Huh7 cells (Fig 1A right panel), suggesting that the EBOV sGP enhances EBOV GP-mediated viral entry in a cell line-specific manner using this assay.

Next, we analyzed the viral entry step in more detail by specifically investigating either attachment or intracellular uptake into HEK293 and Huh7 cells. For this purpose, we produced lipophilic tracer (DiI)-labeled VLPs consisting of the major EBOV structural proteins GP, matrix protein (VP40), and nucleoprotein (NP), and monitored the localization of VLPs in HEK293 or Huh7 cells expressing enhanced green fluorescent protein (eGFP) fused to Rab7 (eGFP-Rab7), a late endosome marker. As a control we used a combination of ZGP12/1.1 plus C1q, which has been shown to induce antibody-dependent enhancement (ADE) of EBOV infection in HEK293 cells [17]. We found no difference in the number of VLPs attached to the cell surface in the presence of CP, EBOV sGP, or RESTV sGP (Figs 1B, 1D and S1A, S1C), suggesting that EBOV sGP does not contribute to increased VLP attachment to the cell surface on both cell lines. In contrast, VLPs treated with ZGP12/1.1 plus C1q significantly enhanced VLP attachment to the cell surface as previously described in HEK293 cells confirming functionality of our assay (Fig 1B and 1D)[17]. The next set of experiments assessed the number of VLPs taken up into intracellular vesicles. The VLP uptake into endosomes was visualized using the HEK293 or Huh7 cells expressing eGFP-Rab7 because of the opportunity of colocalization analysis of eGFP-Rab7 and internalized VLPs these cells provide. We found that the number of intracellular VLPs significantly increased in the presence of EBOV sGP in HEK293 cells (Fig 1C and 1E), and also confirmed the colocalization of the VLPs with Rab7 which is indicative of the VLPs being in late endosomes (Fig 1C and 1F). In contrast, we did not observe a difference in VLP uptake in the presence of EBOV sGP or the other proteins in Huh7-eGFP-Rab7 cells (S1B, S1D and S1E Fig). Taken together these results demonstrated that EBOV sGP does not affect viral attachment to a cell, however, significantly increases the uptake of virus particles into HEK293 cells but not Huh7 cells.

### EBOV sGP increases the replication of EBOV

**Fig 2:**
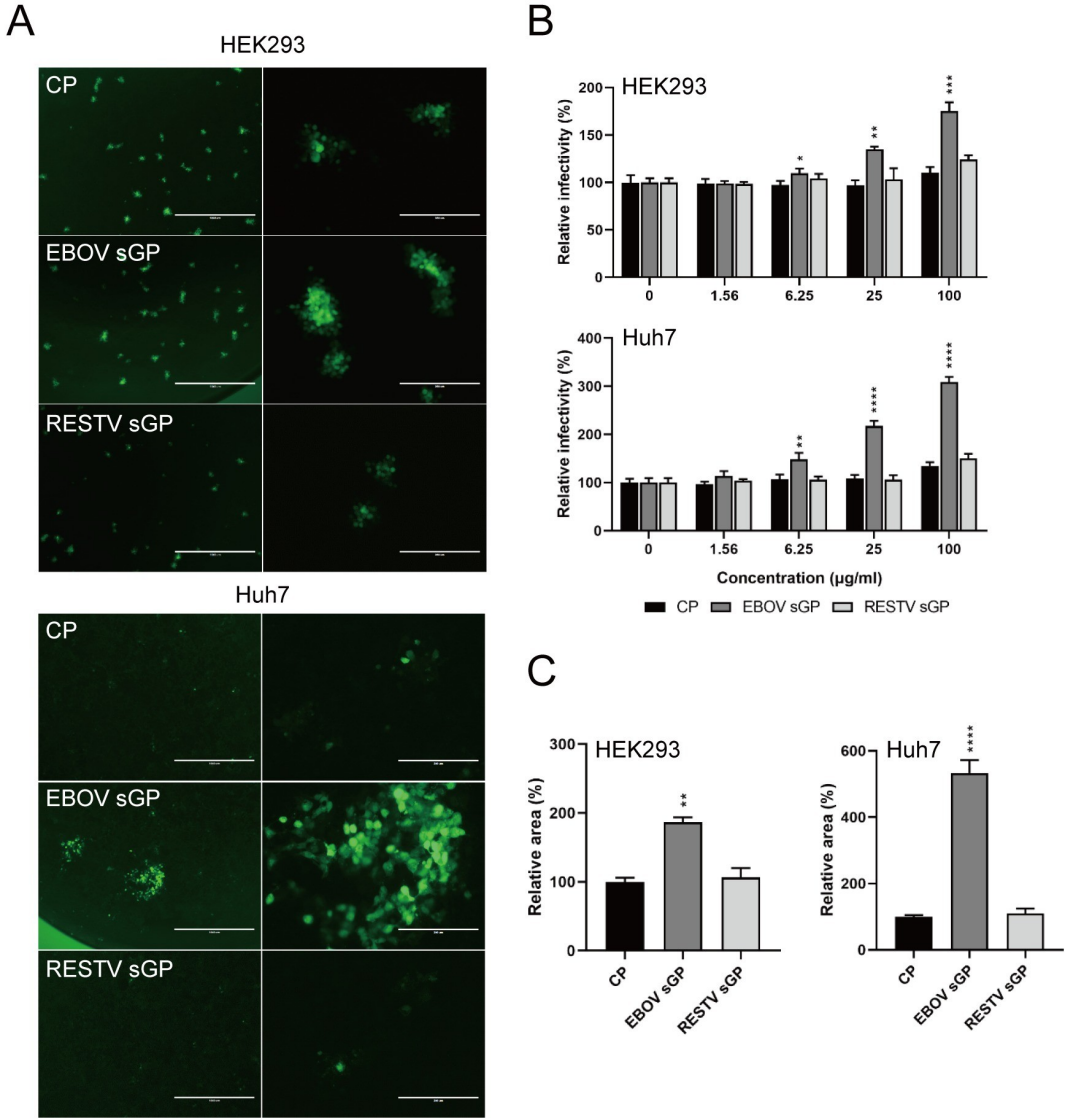
EBOV sGP increases the infectivity of EBOV-GFP. (**A, B, C**) HEK293 and Huh7 cells were infected for 1 h with 50–100 FFU EBOV-GFP and cultured for 72 h in the presence of 100 μg/ml (**A, C**) or the indicated concentration (**B**) of EBOV sGP or RESTV sGP. An equivalent amount of CP was added as a control. (**B**) Scale bars represent 1000 (left), or 250 (right) μm. The relative infectivity and area were calculated by setting the value of each infected cell line without protein (**B**) or with CP (**C**) to 100%. The mean and standard deviation of three independent experiments are shown. Statistically significant differences as determined by one-way ANOVA are indicated as ****p < 0.0001, ***p < 0.001, **p < 0.01, and *p < 0.05.

Analysis of the effect of sGP on EBOV infectivity and replication was performed using EBOV-GFP, which encodes the wt editing site. We confirmed that the addition of EBOV sGP increased the infectivity of EBOV-GFP in both HEK293 and Huh7 cells in a dose-dependent manner (Fig 2A and 2B). Additionally, the virus grew bigger plaques in the presence of EBOV sGP compared to RESTV sGP or CP (Fig 2A and 2C). The relative size of a plaque caused by EBOV-GFP in the presence of EBOV sGP is significantly increased compared to the RESTV sGP or CP (Fig 2C). Interestingly, the effect of EBOV sGP was observed in both cell lines and is more prominent in Huh7 cells than HEK293 cells.

**Fig 3:**
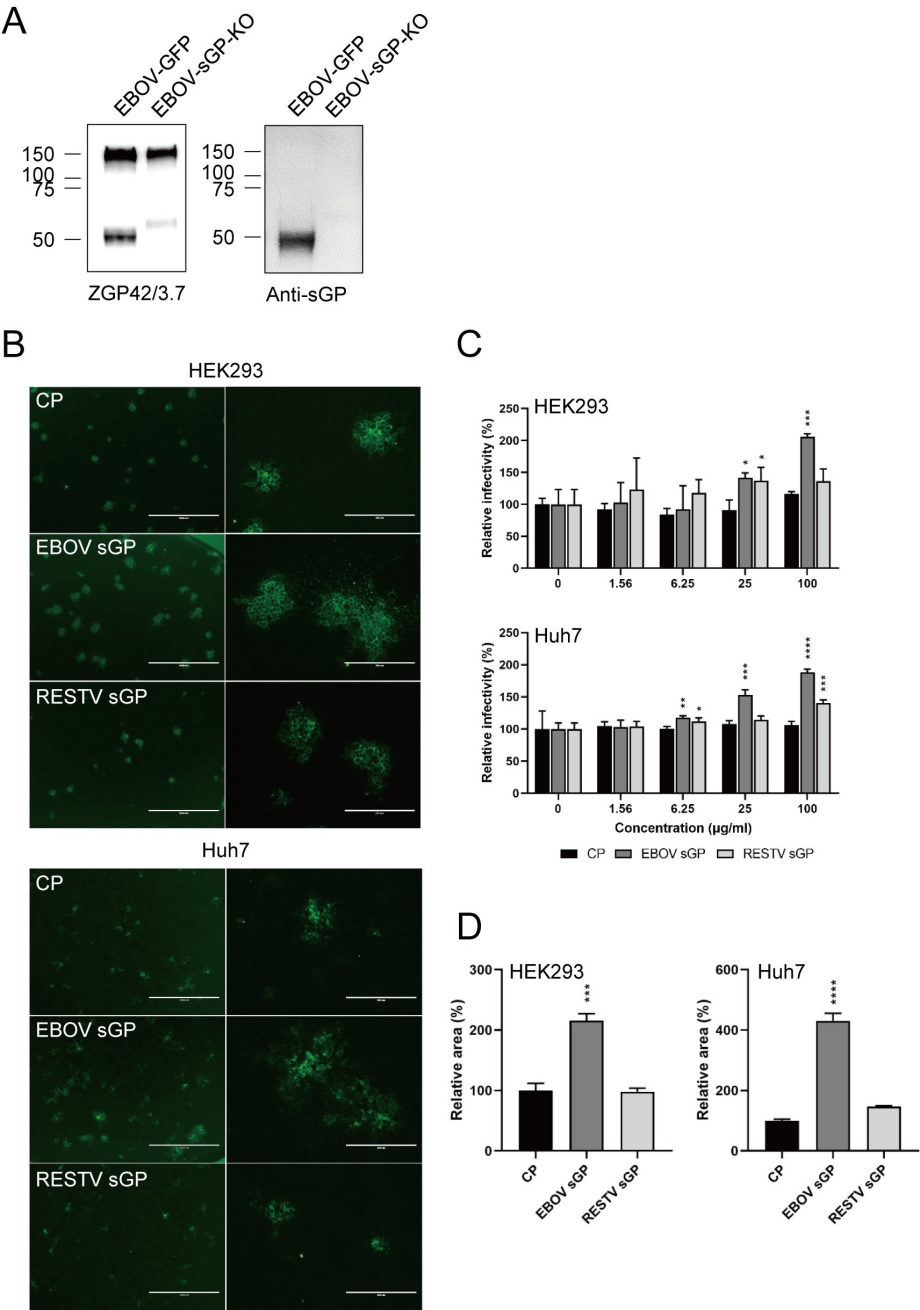
EBOV sGP increases the infectivity of EBOV-sGP-KO. (**A**) Expression of EBOV GP (upper band) and sGP (lower band) EBOV-GFP and EBOV-sGP-KO was confirmed by WB analysis of using an anti-GP monoclonal antibody, ZGP42/3.7 (left; 1 μg/ml) or an anti-sGP polyclonal antibody (right; 1 μg/ml). (**B, C, D**) HEK293 and Huh7 cells were infected for 1 h with 50–100 FFU EBOV-sGP-KO and cultured for 72 h in the presence of 100 μg/ml (**B, D**) or the indicated concentration (**C**) of EBOV sGP, RESTV sGP, or the equivalent amount of CP. (**B**) Scale bars represent 1000 (left), or 250 (right) μm. The relative infectivity and area were calculated by setting the value of each infected cell line without protein (**C**) or with CP (**D**) to 100%. The mean and standard deviation of three independent experiments are shown. Statistically significant differences as determined by one-way ANOVA are indicated as ****p < 0.0001, ***p < 0.001, **p < 0.01, and *p < 0.05.

Further analysis of the effect of EBOV sGP on EBOV replication was performed using EBOV-sGP-KO, a recombinant EBOV with the GP gene mutated to not express sGP [16]. We confirmed the lack of sGP expression by WB using the mouse monoclonal antibody ZGP42/3.7, which detects both GP and sGP. Surprisingly, a faint band approximately the size of sGP was detected in the EBOV-sGP-KO lane (Fig 3A left panel), however, WB analysis with a polyclonal antibody specific to EBOV sGP confirmed that there was no sGP in the EBOV-sGP-KO sample (Fig 3A right panel). We previously established a sandwich ELISA to capture EBOV sGP. Using this assay, we did not detected any sGP in the supernatant of EBOV-sGP-KO-infected cells [16]. These results confirm that the GP mutation in the virus is stable and that there is no expression of sGP. When we infected both cell lines (HEK293 and Huh7) with EBOV-sGP-KO and treated them concomitantly with EBOV sGP, RESTV sGP or CP, infectivity of EBOV-sGP-KO was significantly increased in the presence of EBOV sGP in a dose-dependent manner (Fig 3B and 3C). Interestingly, RESTV sGP also increased the infectivity of EBOV-sGP-KO, however, the effect was lower than that of the EBOV sGP and not consistently dose-dependent (Fig 3B and 3C). In addition, we confirmed that the relative infectious focus size in the presence of EBOV sGP was significantly increased compared to those in the presence of RESTV sGP and CP (Fig 3B and 3D). Finally we perfomed growth kinetics with EBOV or EBOV-sGP-KO in the presence of CP, EBOV sGP and RESTV sGP on Huh7 cells (MOI 0.01). Only EBOV sGP treatment resulted in a significant incresae of virus titers at 48 and 72 h for EBOV or EBOV-sGP-KO, respectively (S2 Fig). These results indicate that EBOV sGP promotes an increase in viral infectivity.

### EBOV sGP activates phosphorylation of MEK1/2 in Huh7 cells

**Fig 4:**
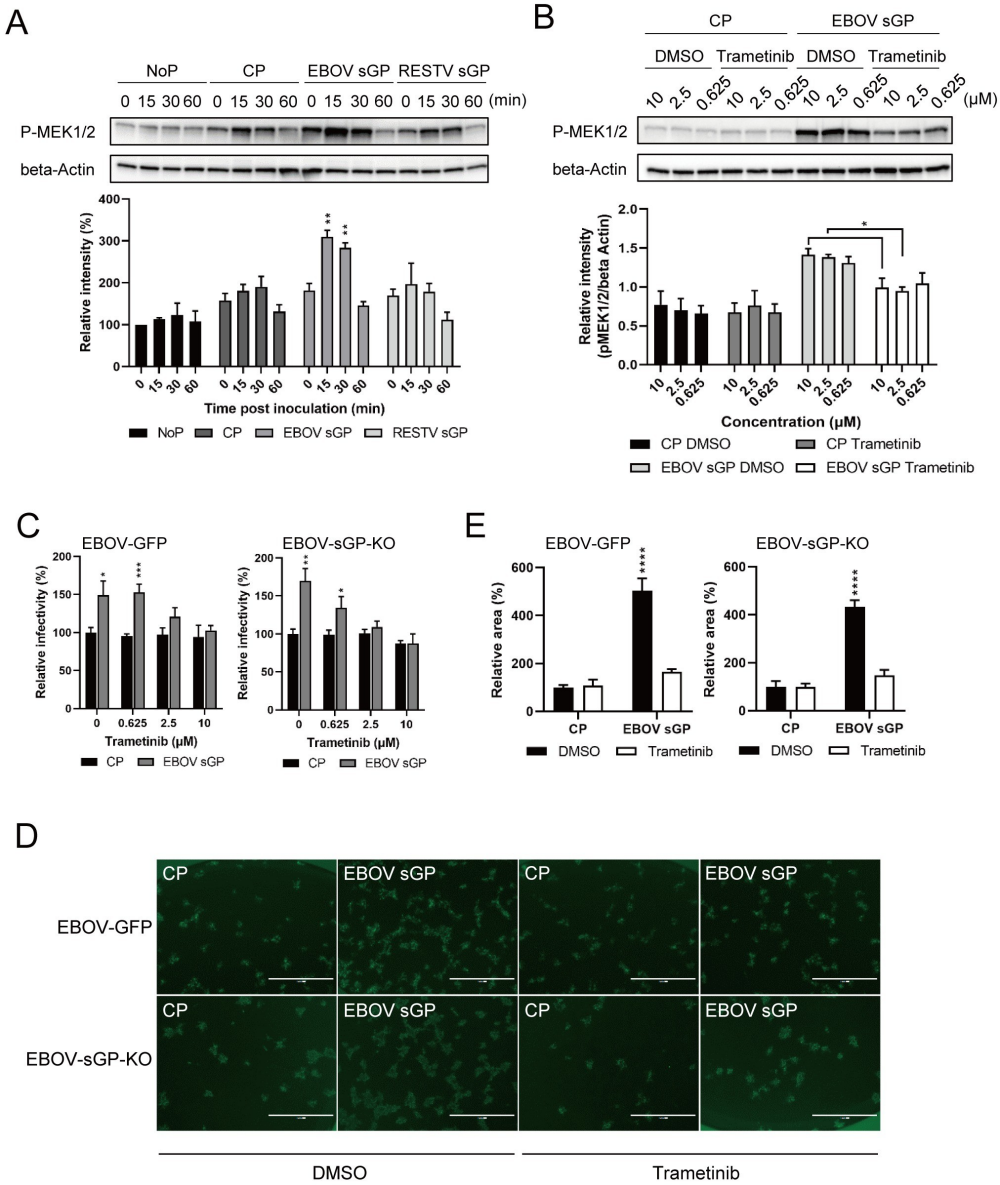
EBOV sGP increases phosphorylation of MEK1/2 and EBOV infectivity in Huh7 cells. (**A**) Huh7 cells were incubated with 100 μg/ml EBOV sGP, RESTV sGP, or the equivalent amount of CP. After 0, 15, 30, and 60 min incubation at 37°C, phosphorylation of MEK1/2 at Ser217/221 (p-MEK1/2) was assessed by WB analysis. The intensity of the p-MEK1/2 band was normalized to that of the β-actin band at each time point. Relative intensities of the p-MEK1/2 bands were then calculated by setting the intensity of cells in the absence of protein (NoP) (0 h) to 100%. (**B**) Huh7 cells were pretreated with 0.625, 2.5 or 10 μM of DMSO or Trametinib for 1 h at 37°C. Then the cells were treated with 100 μg/ml EBOV sGP, RESTV sGP, or equivalent amount of CP. After 15 min at 37°C, phosphorylation of MEK1/2 was detected by WB analysis. The intensity of the p-MEK1/2 band was normalized to that of the β-actin band for each tested condition. Huh7 cells were pretreated with (**C**) the indicated concentration or (**D, E**) 10 μM of Trametinib or the equivalent amount of DMSO for 1 h and infected for 1h with 50–100 FFU EBOV-GFP or EBOV-sGP-KO in the presence of 100 μg/ml of EBOV sGP or the equivalent amount of CP and cultured for 72 h. (**D**) Scale bars represent 1000 μm. The relative infectivity and area were calculated by setting the value of each infected cell with CP in the presence of no inhibitor (**C**) or DMSO (**E**) to 100%. Statistically significant differences as determined by one-way ANOVA (A, B) and two-tailed unpaired *t*-test (C, E) are indicated as ****p < 0.0001, ***p < 0.001, **p < 0.01, and *p < 0.05.

A previous study demonstrated that the robustness of EBOV protein synthesis and replication is affected by activating the MAPK pathway [18]. While HEK293 cells showed a significant increase in virus entry using VSV-EBOV GP, Huh7 cells did not (Fig 1A). However, both cell lines demonstrated a replication increase for EBOV in the presence of EBOV sGP indicating that the differences could be due to the assay system used or they might be tissue-specific. In order to decipher if the activation of this signaling pathway is indeed tissue-specific, we set out to quantify the phosphorylation levels of MEK1/2, a member of the MAPK family and a component of the MAPK signaling cascade in both cell lines [19]. We found that MEK1/2 is stably expressed over time in both cell lines. However, in the presence of CP, EBOV sGP or RESTV sGP, phosphorylation of MEK1/2 occurs at a significantly higher rate in the presence of EBOV sGP in Huh7 cells only (S3A Fig). To investigate this further, we quantified the phosphorylation of MEK1/2 in Huh7 cells with either EBOV sGP, RESTV sGP, CP, or no protein (NoP). Interestingly, the relative band intensity for phosphorylated MEK1/2 increased in all protein-treated cells at 0 minutes (min) (Fig 4A), suggesting that any protein may trigger the phosphorylation of MEK1/2. Notably, there is no significant difference in the RESTV sGP-treated cells compared to CP-treated cells at any time point (Figs 4A and S3A). However, we could show a significant increase of MEK1/2 phosphorylation at 15 and 30 min after Huh7 cells were treated with EBOV sGP (Figs 4A and S3A). This observation suggests that the MAPK signaling pathway is indeed activated by EBOV sGP and may play a key role in the ability of sGP to modulate EBOV replication in Huh7 cells.

In order to strengthen our data pointing towards the MAPK signaling pathway as a contributor to EBOV infectivity in Huh7 cells, we analyzed the EBOV sGP effect in the presence of a MEK1/2 inhibitor called Trametinib. We confirmed by WB analysis that Trametinib indeed decreased the phosphorylation of MEK1/2 induced by EBOV sGP in Huh7 cells (Figs 4B and S3B). Next, we infected Huh7 cells with EBOV-GFP and EBOV-sGP-KO in the presence of different concentrations of Trametinib plus 100 μg/ml EBOV sGP or CP. The addition of EBOV sGP increased the infectivity (numbers of foci) of both EBOVs as previously shown (Figs 2 and 3), and Trametinib countered this effect in a dose-dependent manner (Fig 4C). Similarly, the foci size in infected cells in the presence of EBOV sGP without the drug was increased as previously shown (Figs 2 and 3), and the addition of 10 μM Trametinib eliminated this difference (Fig 4D and 4E). We also examined whether Trametinib increased the infectivity of VSV-EBOV GP in the presence of EBOV sGP in HEK293 cells and found no significant difference between Tametinib and DMSO treatment (S4 Fig). We excluded that treatment with any sGP or Trametinib impacted cell viability (S5 Fig). This data suggests that the EBOV sGP effect in Huh7 cells, namely increased infectivity and replication documented by increased foci numbers and size, is mediated through the MAPK signaling pathway.

### EBOV sGP induces higher EBOV replication in mouse tissues

**Fig 5:**
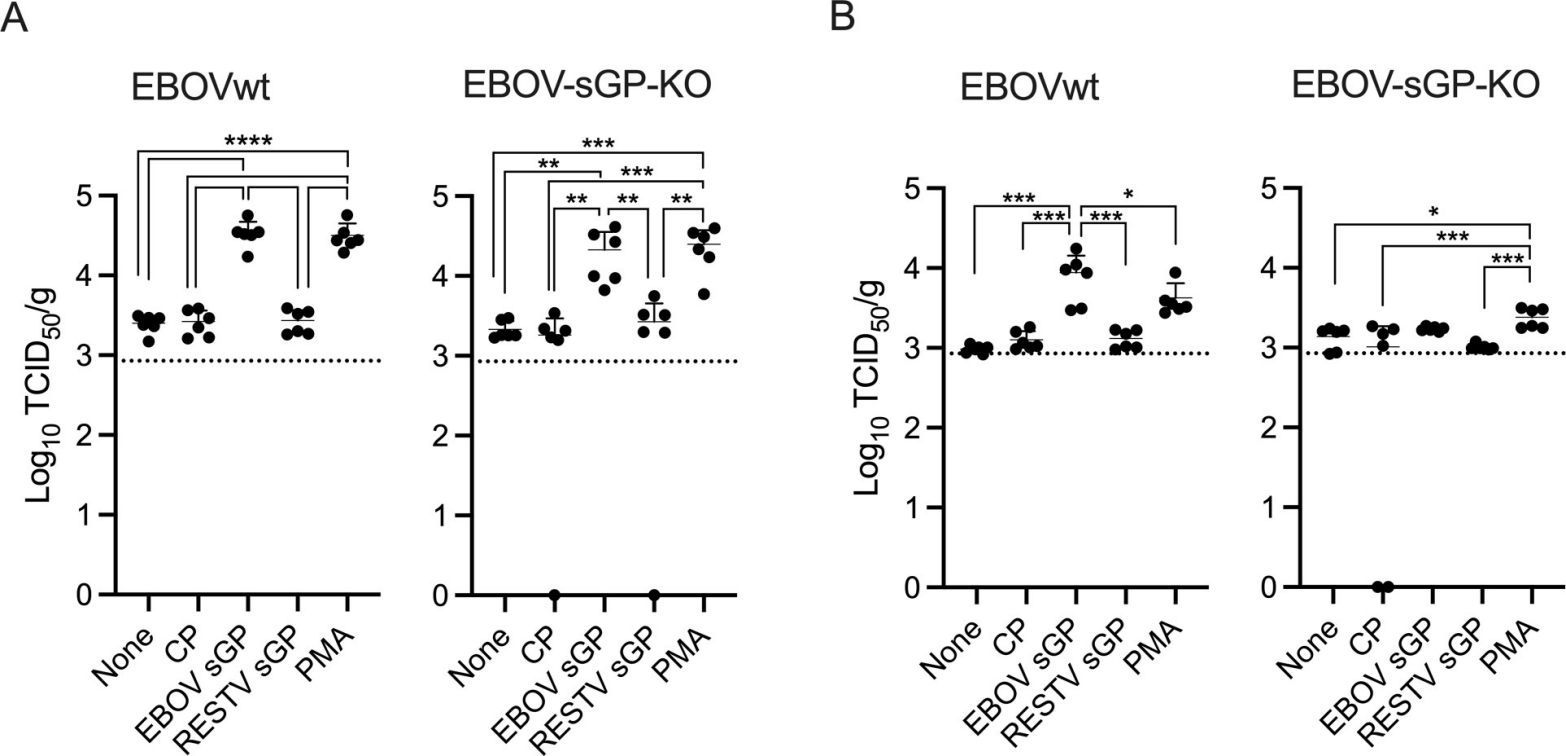
EBOV sGP increases EBOV replication in target tissues of BALB/c mice. Female BALB/c mice (n = 6) were infected with 1,000 FFU of either EBOV or EBOV-sGP-KO. After 6 days, mice received an intraperitoneal injection containing DMEM (None), DMEM containing 200 μg of EBOV sGP, RESTV sGP, the equivalent amount of CP, or 100 ng/ml of PMA. After 24 h, the mice were euthanized, liver and spleen samples were collected, and titers were determined. Geometric mean and standard deviation are shown. Statistically significant differences as determined by one-way ANOVA are indicated as **** *p* < 0.0001, *** *p* < 0.001, ** *p* < 0.01, and *p < 0.5

Finally, and to test if the aforementioned effects of sGP play a role during infection *in vivo*, BALB/c mice were intraperitoneally (i.p.) infected with EBOV wild type (EBOVwt) or EBOV-sGP-KO. The animals were observed daily and did not show signs of disease. Six days later, the mice were stimulated with a single i.p. injection of 200 μg CP, EBOV sGP, or RESTV sGP. As a positive control, we used phorbol-12-myristate-13-acetate (PMA) since a previous study demonstrated that this stimulation activates the MAPK signaling pathway and resulted in higher EBOV organ titers [18]. After 24 h, all mice were euthanized, and liver, spleen and blood samples were collected for EBOV titration. High virus titers were detected in the livers of mice stimulated with EBOV sGP in both the EBOVwt- and EBOV-sGP-KO-infected mice (Fig 5A). The liver titers matched the ones obtained from mice treated with the MAPK activator PMA (Fig 5A). Liver EBOV loads from CP- and RESTV sGP-treated or untreated mice were similar and significantly lower (Fig 5A). Similarly, EBOVwt titers in the spleen were significanyly higher when EBOV sGP treatment occurred, however, not as high as liver titers (Fig 5B). The effect was overall lower with EBOV-sGP-KO infection in the spleen compared EBOVwt infection with EBOV sGP treatment resulting in a slight but not significant increase in titers (Fig 5B). No virus was isolated from blood samples of any of the infected mice. This result highlights the ability of EBOV sGP to enhance EBOV replication in mouse target tissues like the liver and spleen.

## Discussion

Understanding the role of sGP during EBOV infection is important for pathogenesis and may lead to the identification of new therapeutic targets. Previous studies have shown that EBOV sGP may be playing a role as a decoy antigen, a modulator of the immune response or a pathogenicity factor [15,20]. However, these roles that have been suggested for EBOV sGP still require more investigation. In this study, we focused on the effects of sGP during EBOV infection in two cell lines derived from distinct human organs, HEK293 (kidney) and Huh7 (liver). We identified cell-specific novel mechanisms mediated by EBOV sGP leading to increased EBOV replication.

First, we focused on two different human-derived cell lines, HEK293 and Huh7 cells, and found that the effect of EBOV sGP on EBOV replication is indeed different between the two (Figs 1, 2 and 3). Interestingly, EBOV sGP increased the infectivity of VSV-EBOV GP pseudoparticles in HEK293 cells, but not Huh7 cells, suggesting that EBOV sGP might play a role in the early steps of infection in HEK293 cells. Further analysis demonstrated that EBOV sGP indeed increased the uptake of DiI-labeled VLPs into late endosomes in HEK293 cells (Fig 1C and 1E) but nor Huh7 cells (S1B and S1D Fig). Since EBOV sGP binds to the surface of several cells such as granulocytic cells and neutrophils [21], sGP might also bind to a (so far unknown) molecule on HEK293 cells and promote the uptake of EBOV into these cells. It is also possible that our studies using VSV-EBOV pseudoparticles show the limitations of this surrogate system. We readily detect a difference in HEK293 but not Huh7 cells using pseudoparticles and VLPs in entry assays, yet when using live EBOV variants generated by reverse genetics in replication assays, the differences disappear or even revert. This observation requires further studies comparing in detail the initial steps of infection namely attachment, entry and membrane fusion between surrogate systems and live EBOV.

While the EBOV entry step was not affected by the addition of EBOV sGP in Huh7 cells (Figs 1 and S1), EBOV grew bigger plaques in the presence of sGP (Figs 2 and 3) suggesting that this protein might contribute to a later step of EBOV infection, such as replication, transcription, or budding in these cells. Interestingly, the higher concentration (100 μg/ml) of RESTV sGP also increased EBOV-sGP-KO infectivity in Huh7 cells, however, the plaque size was not significantly bigger than that of CP (Fig 3C and 3D). Similarly, replication kinetics were not affected by the addition of RESTV sGP for both viruses tested (S2 Fig). The amino acid identity of EBOV and RESTV sGP is ~60%, thus, the high concentration of RESTV sGP may affect the infectivity of EBOV-sGP-KO similar to EBOV sGP (Fig 3). Additional studies are needed in order to decipher a potential mechanism behind these observations. However, we investigated if EBOV sGP alone has an effect on innate immune pathways and found that EBOV sGP indeed activates the MAPK signaling cascade (MEK1/2), which in turn contributes to increased EBOV infectivity and the formation of larger plaques (Fig 4). The MAPK signaling pathway has been shown to affect the replication of many RNA viruses [19,22], and similarly impacts the EBOV life cycle [18,22]. It is known that MAPK activity is implicated in EBOV VP40-mediated budding [23], and EBOV GP-mediated cytokine production in dendritic cells [24]. Moreover, EBOV VP24 impairs MAPK activation in a cell type-specific manner and blocks the induction of inflammatory responses [25]. Our present study indicates that in addition to these proteins, EBOV sGP also interacts with MAPK resulting in increased EBOV replication. This effect can be blocked by the inhibitor Trametinib, suggesting that it is indeed specific to the MAPK pathway.

Finally, we wanted to analyze if we can detect an effect of EBOV sGP treatment *in vivo* and analyze if there could be a correlation to the MAPK pathway. We followed an experimental strategy published by Strong and colleagues where mice were infected with EBOV and treated after 6 days with PMA [18]. In order to determine the optimal EBOV sGP concentration for treatment, we relied on previous reports that showed that EBOV sGP can be readily detected in the blood of EBOV-infected patients and animals in large quantities [16,26]. Based on this data, we determined that 200 μg are an appropriate concentration of sGP for the treatment EBOV-infected mice for 24 hours in order to determine if EBOV titers increase. We focused on the liver and spleen as early targets during infection and because Huh7 cells are a liver-derived cell line and we saw the biggest impact of sGP on EBOV replication in these cells. We found that treatment with EBOV sGP indeed increased the EBOV titer in the liver (Fig 5A), similar to what has been described and repeated here with PMA [18]. A similar but less pronounced effect was observed in spleen samples (Fig 5B). These results suggest that EBOV sGP increases virus replication by interacting directly or indirectly with the MAPK signaling pathway. Interestingly, the effect of EBOV sGP was shown to be higher in EBOV-infected mice than in EBOV-sGP-KO infected mice (Fig 5). This may depend on the fact that we do not know the replication kinetics of both viruses in BALB/c mice as they do not cause disease or weight loss. We can only speculate that it could be that there is more EBOV in the liver of these mice after 6 days compared to EBOV-sGP-KO. The higher increase in EBOV titer could also be due to the fact that there is already some sGP made by EBOV in the infected mice and the sGP concentration is higher compared to EBOV-sGP-KO-infected mice. More studies are needed in order to confirm these hypotheses.

In conclusion, our data indicate that EBOV sGP has at least two distinct novel functions dependent on the cell type; increasing the uptake and internalization of EBOV virions into intracellular vesicles in HEK293 cells and enhancing EBOV replication by directly or indirectly activating the MAPK signaling pathway in Huh7 cells. Furthermore, our study demonstrated an enhancing effect of EBOV replication in the liver *in vivo*. Taken together with previous findings, EBOV sGP might have different primary functions during the course of infection. After the initial infection of target cells like dendritic cells and macrophages [27], sGP might facilitate and enhance initial spread in the early target tissue like the liver as shown here in Huh7 cells and mice. Later on, EBOV sGP accumulates in the serum at high concentrations [16] and plays a role in vascular dysfunction [28] and as an antibody decoy [15]. Future studies will expand upon this data set and will address outstanding questions like EBOV sGP concentrations in tissues over time during infection and the severity of pathogenicity of EBOV infection with and without sGP. The mechanism behind the activation of the MAPK pathway will also be investigated. We hope to fill in the puzzle pieces regarding the variety of EBOV sGP functions and its role in EBOV pathogenicity.

## Materials and methods

### Ethics statement

All infectious work was performed at the required containment level at the Integrated Research Facility, Rocky Mountain Laboratories (RML), Division of Intramural Research (DIR), National Institute of Allergy and Infectious Disease (NIAID), National Institutes of Health (NIH) according to standard operating protocols approved by the RML Institutional Biosafety Committee. The animal work was approved by the RML Institutional Animal Care and Use Committee (IACUC) and performed according to the guidelines of the Association for Assessment and Accreditation of Laboratory Animal Care, International and the Office of Laboratory Animal Welfare. All procedures on animals were carried out by trained and certified personnel following standard operating procedures (SOPs) approved by the Institutional Biosafety Committee (IBC). Humane endpoint criteria in compliance with IACUC-approved scoring parameters were used to determine when animals should be humanely euthanized.

### Cells and viruses

African green monkey kidney (Vero E6), Human embryonic kidney (HEK) 293, and human hepatoma (Huh) 7 cells were grown in Dulbecco’s modified Eagle’s medium (DMEM) (Sigma-Aldrich) containing 2% or 10% fetal bovine serum (FBS), 2 mM L-glutamine, 50 U/ml penicillin, and 50 μg/ml streptomycin (all from Thermo Fisher Scientific). EBOVwt, recombinant EBOV expressing GFP (EBOV-GFP) [29], and recombinant EBOV-sGP-KO [16] (all based on the EBOV Mayinga isolate) were propagated in Vero E6 cells and stored at -80°C. Titration was performed by focus-forming assay on Vero E6 cells. Replication-incompetent VSV pseudotyped with EBOV GP containing GFP instead of the VSV G gene (VSV-EBOV GP) was generated as described previously [7,30]. Virus titers were determined on Vero E6 cells.

### Cell culture assays

Cell viability was assessed by trypan blue staining. EBOV sGP and RESTV sGP were generated and concentrated as described previously [16]. The supernatant of untransfected cells was also concentrated using the same method [16] and used as the control protein (CP). EBOV-GFP and EBOV-sGP-KO were appropriately diluted to provide 50–100 focus-forming units (FFU)/25 μl in HEK293 or Huh7 cells and mixed with EBOV sGP, RESTV sGP (100, 25, 6.25, 1.56 μg/ml) or an equivalent amount of CP. Confluent HEK293 or Huh7 cells cultured in 96-well plates were inoculated with EBOV alone, EBOV/CP or EBOV/sGP mixtures and incubated for 1 h at 37°C. After adsorption, the inoculum was replaced with Eagle’s minimal essential medium (EMEM) containing 1.2% carboxymethyl cellulose and EBOV sGP, RESTV sGP (100, 25, 6.25, 1.56 μg/ml), or an equivalent amount of CP. After incubation for 3 days, cells were fixed with 10% phosphate-buffered formalin. EBOV-sGP-KO-infected cells were immunostained with a mouse anti-EBOV GP monoclonal antibody (ZGP12/1.1, kindly provided Ayato Takada, Hokkaido University, Japan) as primary antibody followed by anti-mouse IgG conjugated with Alexa Fluor 488 (Thermo Fisher Scientific) as a secondary antibody. Titers of EBOV-GFP or EBOV-sGP-KO were quantified by counting the number of fluorescent foci. Imaging was performed using EVOS FL Cell Imaging System (Thermo Fisher Scientific) and foci size was determined using ImageJ software (NIH). VSV-EBOV GP appropriately diluted to yield 50–100 infectious units (IUs)/50 μl in HEK293 or Huh7 cells was inoculated with each protein into HEK293 or Huh7 cells. GFP-positive cells were counted after 24 h.

### Western blot analysis

Recombinant EBOVs were mixed 1:1 with sodium dodecyl sulfate-polyacrylamide (SDS) gel electrophoresis sample buffer containing 20% β-mercaptoethanol (Sigma) and heated to 99°C for 10 min. Samples were removed from the maximum containment laboratory following IBC-approved SOPs. SDS-PAGE with all samples was performed in parallel on TGX criterion pre-cast gels (Bio-Rad Laboratories) and proteins were transferred to a Trans-Blot polyvinylidene difluoride membrane (Bio-Rad Laboratories). Protein detection was performed using the mouse monoclonal anti-EBOV GP (ZGP 42/3.7, 1 μg/ml; kindly provided by Ayato Takada, Hokkaido University, Sapporo, Japan) or rabbit polyclonal anti-EBOV sGP (IBT bioservices). After horseradish peroxidase (HRP)-labeled secondary antibody staining using anti-mouse IgG (1:10,000) or anti-rabbit IgG (1:5,000) (both Jackson ImmunoResearch) for 1 h at room temperature, the bound antibodies were visualized with SuperSignal West Pico chemiluminescent substrate (Thermo Fisher Scientific) in an iBright CL1500 Imaging System (Thermo Fisher Scientific).

### Imaging of attachment and internalization of lipophilic tracer (DiI)-labeled virus-like particles (VLPs)

For imaging of virus attachment and internalization, we generated, purified and DiI-labeled Ebola VLPs containing GP, VP40, and NP as described previously [31]. HEK293 and Huh7 cells expressing enhanced green fluorescent protein fused to Rab7 (eGFP-Rab7; kindly provided Asuka Nanbo, Nagasaki University, Japan), a late endosome marker, were cultured in 35 mm glass-bottom dishes (MatTek Corporation) precoated with borate buffer containing 0.1 mg/ml poly-L-lysine (Sigma). The cells were inoculated with 1 μg/ml DiI-labeled VLPs in the presence of 100 μg/ml EBOV sGP, RESTV sGP, or an equivalent amount of CP and incubated for 30 min on ice. After adsorption of VLPs, the cells were incubated with DMEM containing 2% FCS and 4% bovine serum albumin (BSA) for 0 or 2 h at 37°C. To count the number of DiI-labeled VLPs, the cells were fixed with 4% paraformaldehyde (Electron Microscopy Sciences) for 30 min at room temperature. The nuclei were stained with ProLong Gold Antifade reagent with DAPI (Thermo Fisher Scientific). Microscopic images were acquired as described previously [17] using a confocal laser-scanning microscope (Zeiss LSM710; Carl Zeiss or FV3000; Olympus) and the ZEN 2012 software (Carl Zeiss) or the FV31S-SW software (Olympus). The number of DiI-labeled VLPs was determined in approximately 50–100 individual cells using Image J software (NIH) and the average number per cell was calculated for each condition. For colocalization analysis, we measured the percentages of DiI-labeled VLPs that colocalized with eGFP-Rab7 (i.e., DiI and eGFP-double positive pixels/total DiI-positive pixels × 100) using the Coloc module in ZEN 2012 software (Carl Zeiss) or Image J software.

### Phosphorylation assay

Huh7 or HEK293 cells were incubated with 100 μg/ml of EBOV sGP, RESTV sGP, or the equivalent amount of CP for 0, 15, 30, or 60 min at 37°C. At each time point, cells were collected and washed once in PBS then suspended in PBS. Next, the samples were mixed with SDS gel electrophoresis sample buffer containing 20% β-mercaptoethanol (Sigma) and boiled for 10 min. The samples were electrophoresed by SDS-PAGE and transferred to a Trans-Blot polyvinylidene difluoride membrane as described above. The membrane was incubated with a phospho-MEK1/2 (Ser217/221) (Cell Signaling), MEK1/2 (Cell Signaling) or β-Actin (Sigma) antibody in PBS containing 1% BSA overnight at 4°C. For visualization, the membrane was incubated with HRP-labeled secondary antibody staining using anti-mouse IgG (1:10,000) or anti-rabbit IgG (1:5000) (both Jackson ImmunoResearch) for 1 h at room temperature. The bound antibodies were visualized with SuperSignal West Pico chemiluminescent substrate (Thermo Fisher Scientific) or Clarity Wester ECL Substrate (Bio Rad) in an iBright CL1500 Imaging System (Thermo Fisher Scientific) or WSE-6100 LuminoGraph I (ATTO), respectively. Band intensities were analyzed with ImageJ software (NIH).

### Inhibitor treatments

For infection inhibition assays, the MEK1/2 inhibitor Trametinib (Focus biomolecules) was used at 0.625–10 μM on Huh7 cells. Cells were treated with Trametinib for 1h at 37°C and then 100 μg/ml EBOV sGP or an equivalent amount of CP for each concentration of Trametinib mixture were added to Huh7 cells. After 15 min incubation at 37°C, samples were collected, and WB analysis was performed as described above. For the sGP assay, Huh7 cells were treated with Trametinib for 1h at room temperature and then removed the supernatant. EBOV with CP or 100 μg/ml EBOV sGP were mixed with each concentration of Trametinib and the mixture was added to Huh7 cells. After 1 h incubation at 37°C, the inoculum was replaced with EMEM containing 1.2% carboxymethyl cellulose, each concentration of Trametinib, and 100 μg/ml EBOV sGP or CP. The cells were then incubated and observed as described above. For the sGP inhibition assay using VSV-EBOV GP, HEK293 cells were treated with Trametinib for 1h at room temperature. VSV-EBOV GP with CP or 100 μg/ml EBOV sGP with each concentration of Trametinib was added to the HEK293 cells. GFP-positive cells were counted after 24 h.

### Animal study outline

Groups of female BALB/c mice (n = 6) were challenged via i.p. injection with 1,000 FFU of either EBOV or EBOV-sGP-KO. On day 6 after challenge, the mice were inoculated i.p. with 200 μl of DMEM containing 200 μg of EBOV sGP, RESTV sGP, the equivalent amount of CP, or 100 ng/ml of PMA. Mice were euthanized 24 h later, and tissue samples were collected for virus titration as described previously [32].

### Statistical analysis

Statistical analysis was performed in Prism 7 (GraphPad). Data was analyzed for statistical significance using either two-tailed unpaired *t*-test, one-way or two-way ANOVA with multiple comparisons. Statistical significance is indicated as follows: **** *p* < 0.0001, *** *p* < 0.001, **p < 0.01, and *p < 0.05.

## Supporting information

S1 Fig Effect of sGP treatment on entry of Ebola VLPs. (PNG)

S2 Fig Effect of sGP treatment on EBOV replication. (PNG)

S3 Fig Western blot analysis of p-MEK1/2 and MEK1/2 in each cell line. (PNG)

S4 Fig Effect of Trametinib on the VSV-EBOV GP infectivity. (PNG)

S5 Fig Cell viability in the presence of EBOVsGP, RESTV sGP or Trametinib. (PNG)
