# Supplementary figures and images for: The Ebola virus soluble glycoprotein contributes to viral pathogenesis by activating the MAP kinase signaling pathway
Source: PLoS Pathog. 2021 Sep 16;17(9):e1009937. doi: 10.1371/journal.ppat.1009937 (PMC8478236; doi:10.1371/journal.ppat.1009937)

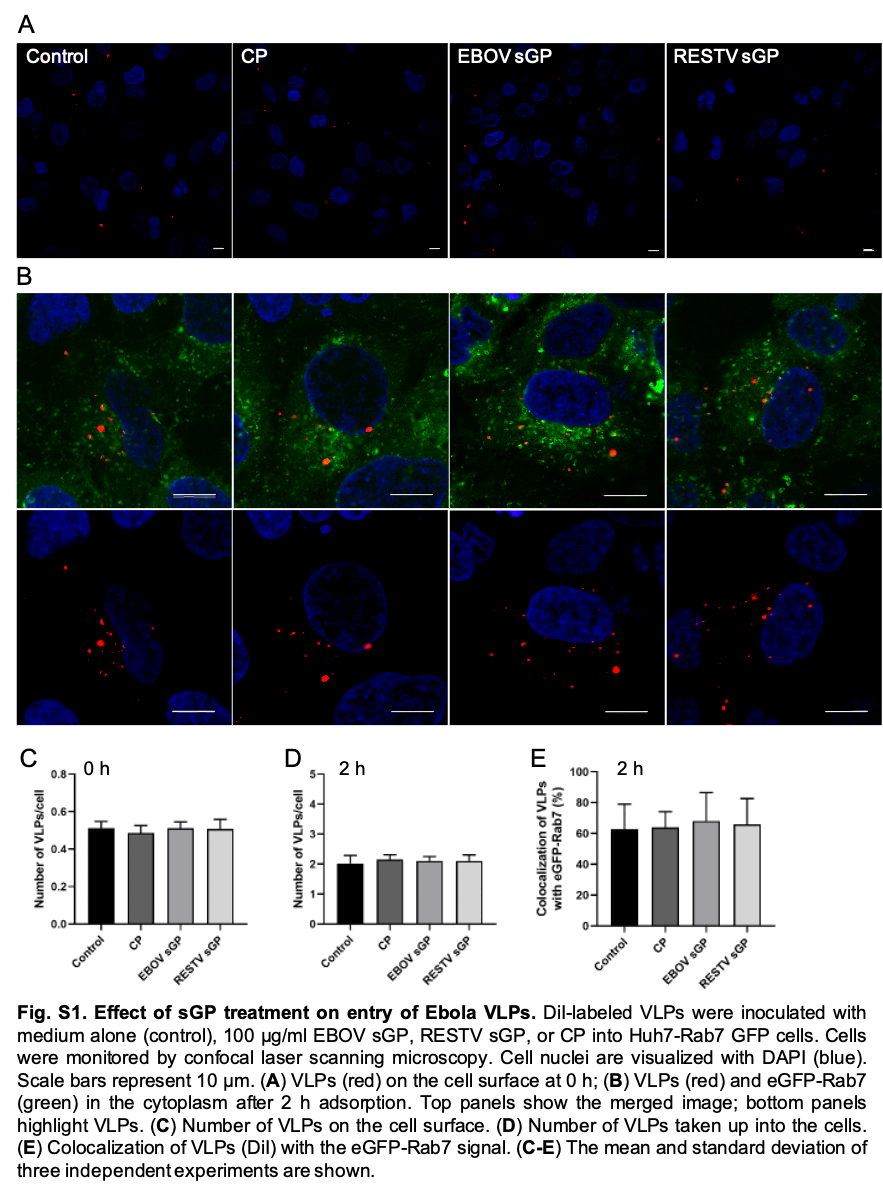

Supplement: S1 Fig — (PNG) [file ppat.1009937.s001.png]

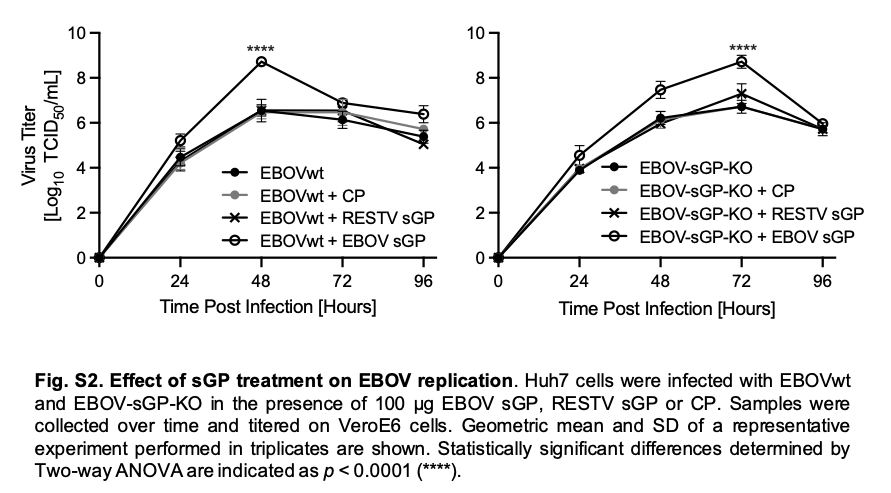

Supplement: S2 Fig — (PNG) [file ppat.1009937.s002.png]

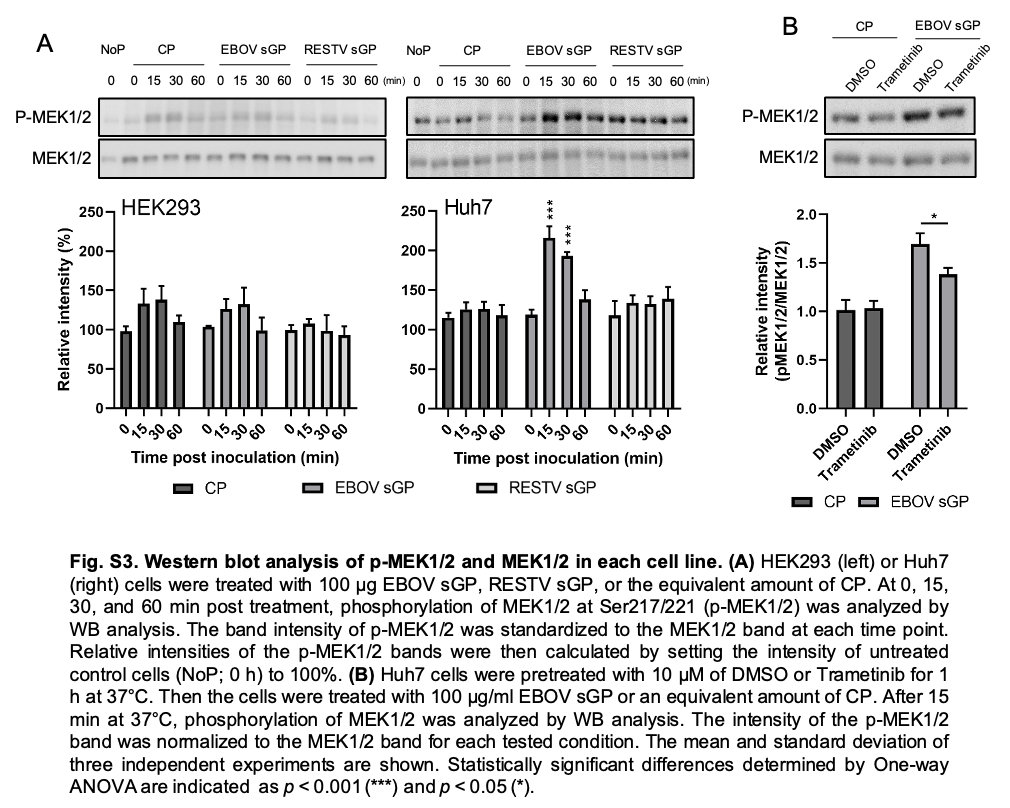

Supplement: S3 Fig — (PNG) [file ppat.1009937.s003.png]

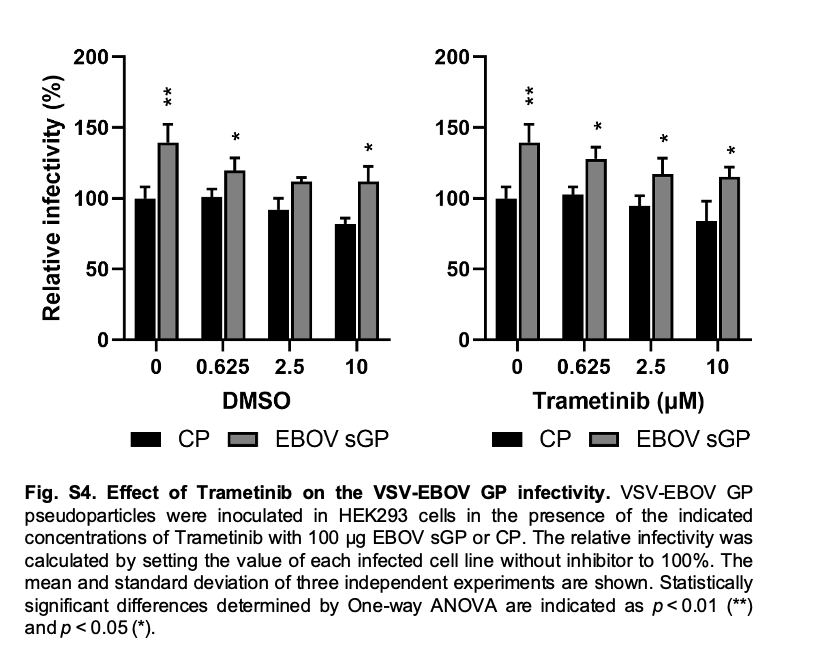

Supplement: S4 Fig — (PNG) [file ppat.1009937.s004.png]

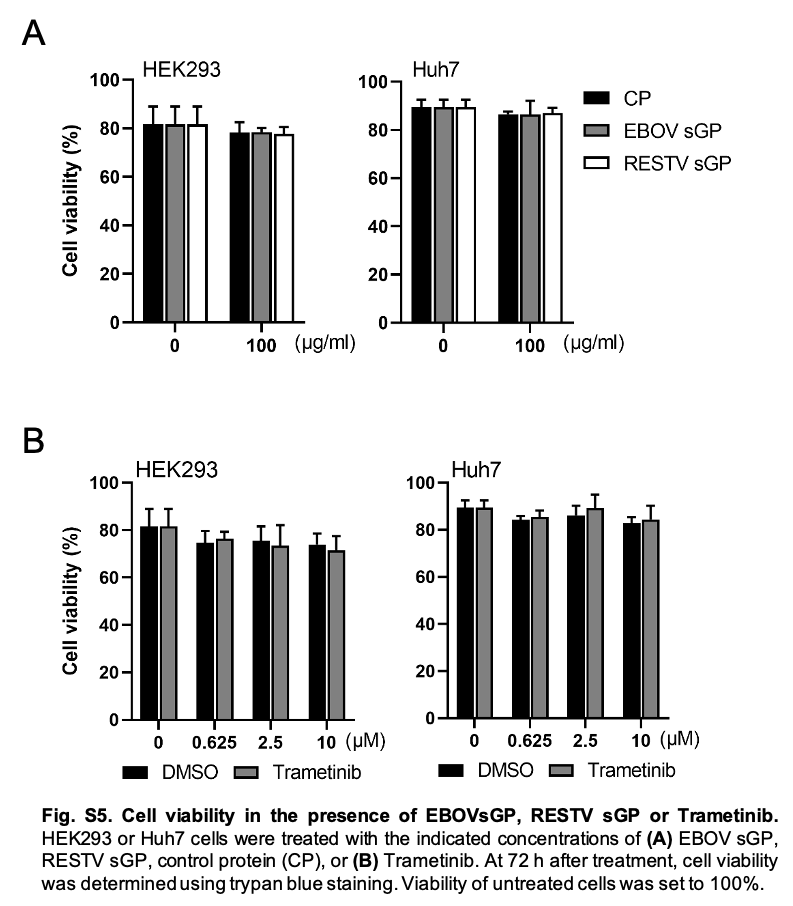

Supplement: S5 Fig — (PNG) [file ppat.1009937.s005.png]
